# Effect of Different Fertilizer Application on the Soil Fertility of Paddy Soils in Red Soil Region of Southern China

**DOI:** 10.1371/journal.pone.0044504

**Published:** 2012-09-28

**Authors:** Wenyi Dong, Xinyu Zhang, Huimin Wang, Xiaoqin Dai, Xiaomin Sun, Weiwen Qiu, Fengting Yang

**Affiliations:** 1 Key Laboratory of Ecosystem Network Observation and Modeling, Institute of Geographic Sciences and Natural Resources Research, Chinese Academy of Sciences, Beijing, People’s Republic of China; 2 The New Zealand Institute for Plant and Food Research Limited, Christchurch, New Zealand; Roehampton University, United Kingdom

## Abstract

Appropriate fertilizer application is an important management practice to improve soil fertility and quality in the red soil regions of China. In the present study, we examined the effects of five fertilization treatments [these were: no fertilizer (CK), rice straw return (SR), chemical fertilizer (NPK), organic manure (OM) and green manure (GM)] on soil pH, soil organic carbon (SOC), total nitrogen (TN), C/N ratio and available nutrients (AN, AP and AK) contents in the plowed layer (0–20 cm) of paddy soil from 1998 to 2009 in Jiangxi Province, southern China. Results showed that the soil pH was the lowest with an average of 5.33 units in CK and was significantly higher in NPK (5.89 units) and OM (5.63 units) treatments (*P*<0.05). The application of fertilizers have remarkably improved SOC and TN values compared with the CK, Specifically, the OM treatment resulted in the highest SOC and TN concentrations (72.5% and 51.2% higher than CK) and NPK treatment increased the SOC and TN contents by 22.0% and 17.8% compared with CK. The average amounts of C/N ratio ranged from 9.66 to 10.98 in different treatments, and reached the highest in OM treatment (*P*<0.05). During the experimental period, the average AN and AP contents were highest in OM treatment (about 1.6 and 29.6 times of that in the CK, respectively) and second highest in NPK treatment (about 1.2 and 20.3 times of that in the CK). Unlike AN and AP, the highest value of AK content was observed in NPK treatments with 38.10 mg·kg^−1^. Thus, these indicated that organic manure should be recommended to improve soil fertility in this region and K fertilizer should be simultaneously applied considering the soil K contents. Considering the long-term fertilizer efficiency, our results also suggest that annual straw returning application could improve soil fertility in this trial region.

## Introduction

Red soils, which can be classified as Ultisols in the Soil Taxonomy System of the USA and Acrisols and Ferralsols in the FAO legend [1], occupy approximately 2.04 million km^2^ in tropical and subtropical regions of China [1]–[3]. In these red soil regions, Rice (*Oryza sativa* L.) is the main cereal crop, contributing 19% and 29% of the world rice area and rice production, respectively [4]. Paddy soil, formed under interchange between drying and wetting rice field conditions, is considered to be the most important soil resource for the food security of China [5]. In recent years, due to the rapid population growth and a continuous decline in the amount of cultivated land area, the rate of fertilizer application keeps on rising in these regions in order to obtain high crop production in agriculture [6]. Nevertheless, instead of improving the soil structure and fertility, the long-term inappropriate fertilization has caused severe degradation of red soils, characterized by high acidity, low nutrients and a disturbed, unbalanced ecosystem [7]. Therefore, how to ameliorate degraded paddy soils in the red soil region and maintain the region’s sustainable development of agricultural production has become an urgent problem.

Recently, soil quality has gained attention as a result of environmental issues related to soil degradation and production sustainability under different farming systems [8]. It has been considered by previous researches that the concentrations of soil nutrients (e.g., organic C, N, P, and K) are good indicators of soil quality and productivity because of their favorable effects on the physical, chemical, and biological properties of soil [9]. Soil pH affects the chemical reactions in soil [10]. Extremes of pH in soils, for example, will lead to a rapid increase in net negative surface charge and thus increases the soil’s affinity for metal ions [11], [12]. Soil organic components, such as soil organic carbon (SOC) or total N (TN) are the most critical indices of paddy soil fertility [13]. Dynamics of SOC and TN storage in agricultural soils drives microbial activity and nutrient cycles, promotes soil physical properties and water retention capacity, and reduces erosion [14]. Moreover, it has been recognized that soil available nutrients (including N, P and K), coming from mineralization and available components of fertilizer, can be directly absorbed by plants, contributing greatly to the soil fertility [15].

With the development of agricultural production, fertilization has been widely used as a common management practice to maintain soil fertility and crop yields [16]. Long-term field experiments (LTFEs) using different agronomic management can provide direct observations of changes in soil quality and fertility and can be predictions of future soil productivity and soil environment interactions [17], [18]. Over past decades, a great number of long-term experiments were initiated to examine the effects of fertilization on soil fertility in the world [19]–[22]. Some studies have documented that the use of fertilizers was necessary, and that continuous fertilizer application increased the concentrations of SOC, TN and other nutrients in plough layers compared with the initial value at the beginning of the experiment [23]–[25]. Manure amendments markedly increased the contents of SOC, TN, and other available nutrients, and reduced soil acidification [13], [26]. However, other studies have shown that the continued use of fertilizers may result in the decline of soil quality and productivity [27], [28]. Long-term application of fertilizer was inadequate to maintain levels of nutrients, the SOC and TN significantly decreasing under the fertilizer treatment and the available N (AN), available P (AP) and available K (AK) did not show clear changes with time or between treatments despite some variation [29], [30]. Thus, there’s still some debate over the effect of different fertilization treatments on soil fertility.

Qianyanzhou experimental station, which was founded in 1982 by Chinese Academy of Sciences (CAS), is noted for its studies on the integrated development and management of natural resources in red earth hilly regions. It has been identified as the red soil hilly system of international experimental demonstration research station in UNESCO’ s programme on Man and the Biosphere (MAB), and now has become an essential station of the Chinese Ecosystem Research Network (CERN) [31]. In paddy soil of southern China, some LTFEs have been initiated [32], [33]. These experiments have provided basic data for research into paddy soil. However, most of them dealt with different chemical fertilizer rates in long-term experiments but few studies focused on different types of fertilizers (such as green manure and rice straw return) affecting the soil fertility. A thorough understanding of how these fertilizers and varying management practices affect the long-term soil fertility of conventional cropping systems is still lacking in Qianyanzhou region, one of the most important typical red earth hill regions in China. In this study, five fertilization treatments (no fertilizer, rice straw return, chemical fertilizer, organic manure and green manure) were applied. Our objectives were to (i) assess the changes of soil fertility parameters in Qianyanzhou from 1998 to 2009 (ii) evaluate the effects of different fertilization treatments on soil fertility parameters, and (iii) put forward suggestions to improve soil fertility in agricultural regions of southern China.

**Figure 1:**
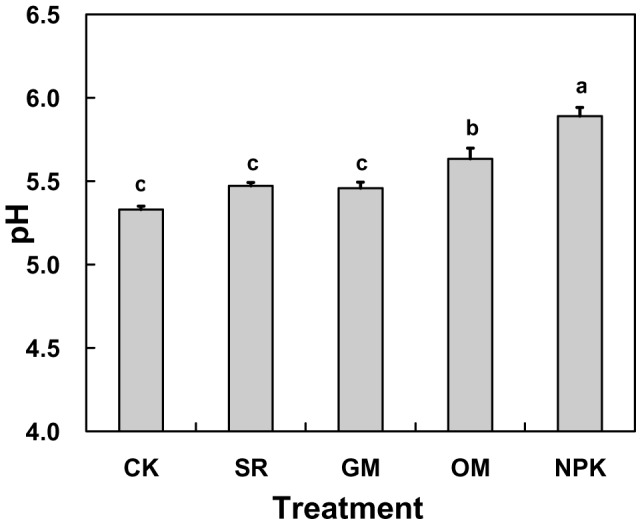
Average soil pH in the different fertilizer treatments. CK: no fertilizer; SR: straw returning rice field; NPK: chemical fertilizer; OM: organic manure and GM: green manure.

## Materials and Methods

### Experimental Site

Qianyanzhou Experimental Station (115°03′29.2″E, 26°44′29.1″N) of CAS is situated on the typical red earth hill region in the mid-subtropical monsoon landscape zone of Taihe County, Jiangxi Province, China. The average elevation is approximately 100 m, and relative relief is 20–50 m. Qianyanzhou Experimental Station has a subtropical monsoon climate. According to the statistics of the meteorological data, the mean annual temperature at this site is 17.8°C, and the annual active accumulated temperatures (above 0 and 10 degrees Celsius) are 6543.8 and 5948.2 degrees Celsius respectively. The annual precipitation and evaporation are 1471.2 mm and 259.9 mm respectively, with the mean relative humidity of 83%. The frost-free period is 290 d and global radiation is 4223 MJ·m^−2^. Our experimental field is located in the flat floodplain where the soil-forming parent material consists of red sandstone and sandy conglomerate. Based on the investigation and analysis before our experiment, it can be concluded that paddy soil is the main soil type with bulk density of 1.50 g·cm^−3^ (0–20 cm), pH of 5.97, soil organic carbon of 9.71 g·kg^−1^, total N content of 1.02 g·kg^−1^, available P content of 1.56 mg·kg^−1^and available K content of 17.61 mg·kg^−1^.

**Figure 2:**
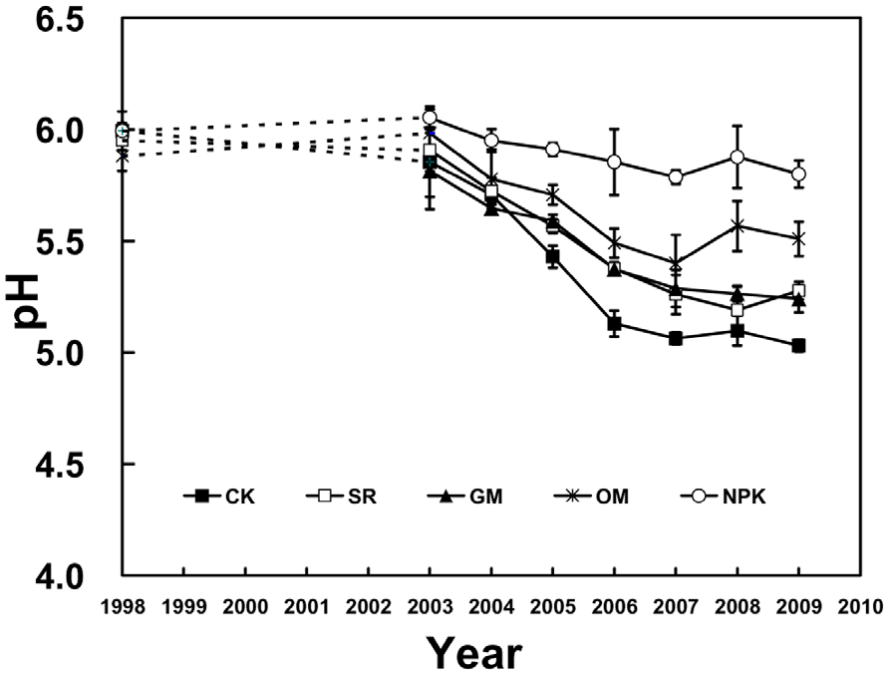
Dynamics of soil pH in the different fertilizer treatments during 1998–2009. Soil samples for 1999–2002 were not analyzed (dashed lines).

**Figure 3:**
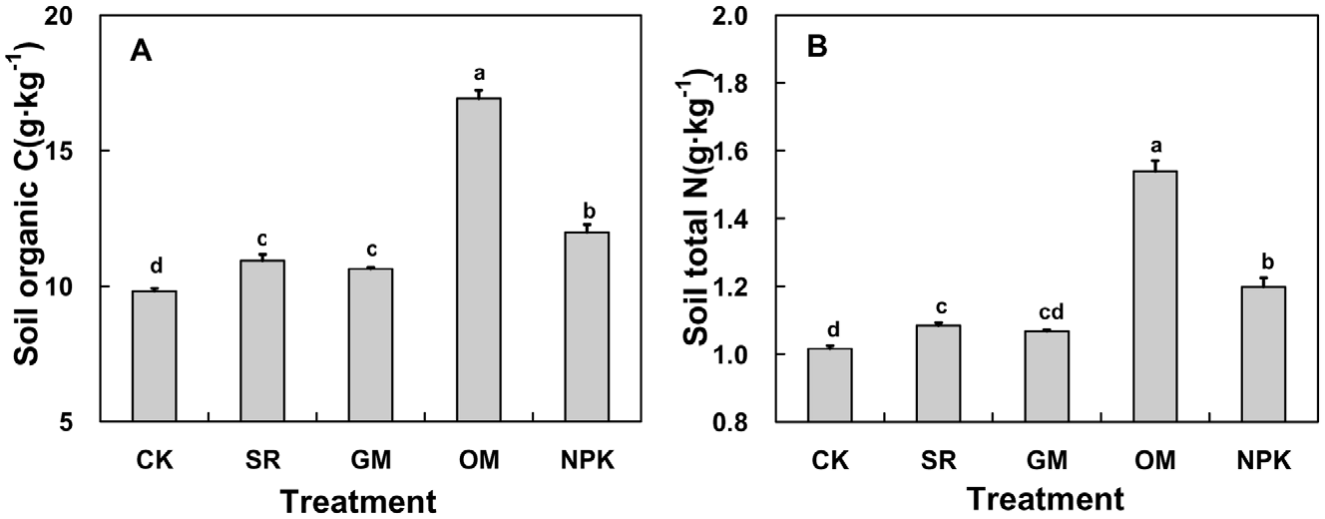
Average SOC and TN in the different fertilizer treatments. CK: no fertilizer; SR: straw returning rice field; NPK: chemical fertilizer; OM: organic manure and GM: green manure.

### Experimental Design

A long-term fertilization experiment was conducted initially in 1998 under a double rice cropping system (rice-rice-winter fallow) which is one of the most common cropping systems in the region. Summer rice was sown at the end of April and harvested in July. Winter rice was sown at the end of July and harvested in November. During the growing season, hand weeding was done to control weeds.

**Figure 4:**
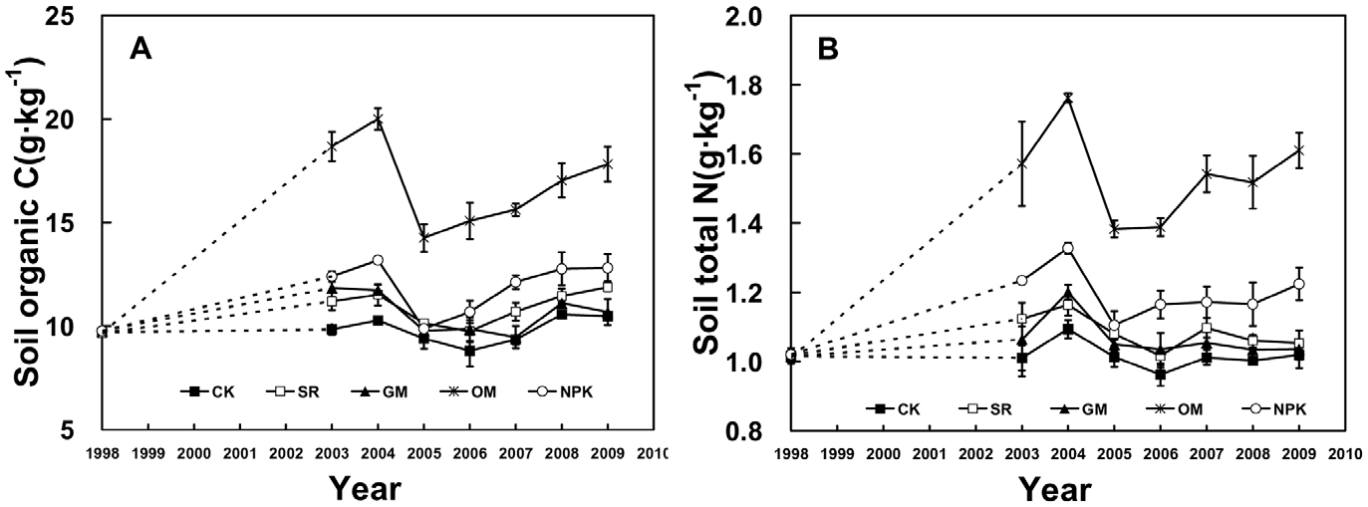
Dynamics of SOC and TN in the different fertilizer treatments during 1998–2009. Soil samples for 1999–2002 were not analyzed (dashed lines).

There were five treatments in total: no fertilizer (CK), straw returning rice field (SR), chemical fertilizer (NPK), organic manure (OM) and green manure (GM). All treatments were arranged in a randomized block design with three replications [25], [34], [35], totalling 15 plots. Each plot was 15 m^2^ (3 m×5 m) and was isolated by concrete walls (50 cm depth and 15 cm above the soil surface). These fertilization systems were chosen based on several common fertilization experiences from local farmers. In SR treatment, all the aboveground rice residues were returned to the soil after harvest, about 4500 kg·hm^−2^ on dry weight. In NPK treatments, inorganic fertilizers were applied at the rates of N-P_2_O_5_-K_2_O at 225–135–225 kg·hm^−2^ by using urea, calcium-magnesium phosphate and potassium chloride. Before sowing, 60% of N, P and K fertilizer were applied as base fertilizers and the remaining fertilizer was applied as top-dressing. In OM treatment, organic manure which came from the faeces of pigs was applied at the rate of 4100 kg·hm^−2^ fresh weight. All pig manure used in our experiment came from a pig farm in Taihe County, where the composting-process was conducted at high temperatures and a good organic fertilizer was obtained after a few months of fermentation by sterilization, deodorization, and so on [36], [37]. In GM treatment, fresh Chinese milk vetch (*Astragalus sinicus* L.) was applied at the rate of 22500 kg·hm^−2^ fresh weight according to the local conventional green manure application rate.

**Figure 5:**
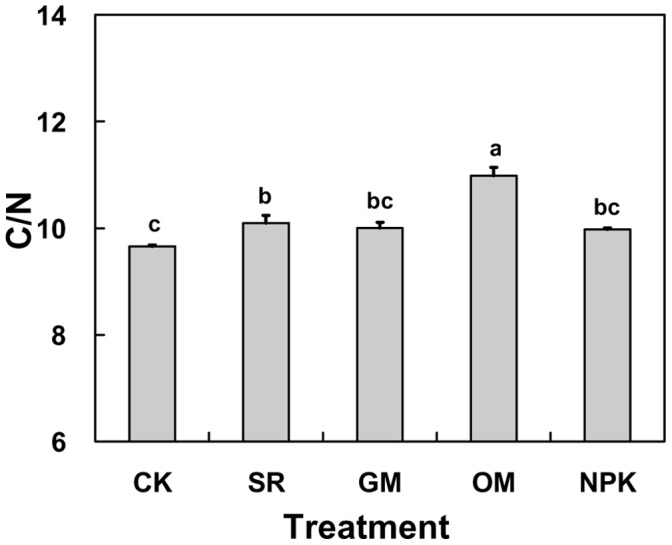
Average C/N ratios in the different fertilizer treatments. CK: no fertilizer; SR: straw returning rice field; NPK: chemical fertilizer; OM: organic manure and GM: green manure.

**Figure 6:**
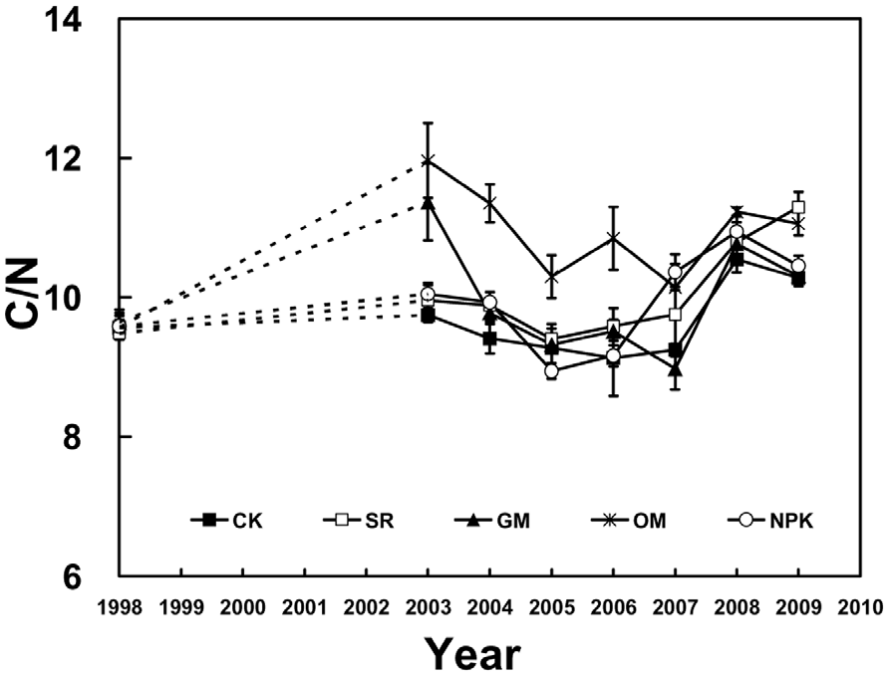
Dynamics of soil C/N ratios in the different fertilizer treatments during 1998–2009. Soil samples for 1999–2002 were not analyzed (dashed lines).

### Soil Sampling and Analysis

Soil samples in the 15 plots were collected annually during 2003–2009 at 7–10 days after the harvest of the late rice. To reflect the real effect of long-term fertilization on the soil fertility, the data in the first 5 years of this experiment were not obtained to analyze in our paper. In each plot, soils were sampled with an auger with 5 cm internal diameter in the plough layer (0–20 cm) at five randomly selected locations and then mixed as one sample [35], [38], [39]. All fresh soil samples were air-dried and sieved through a 2.0 mm and 0.25 mm sieve and stored for nutrient analysis [38], [39].

**Figure 7:**
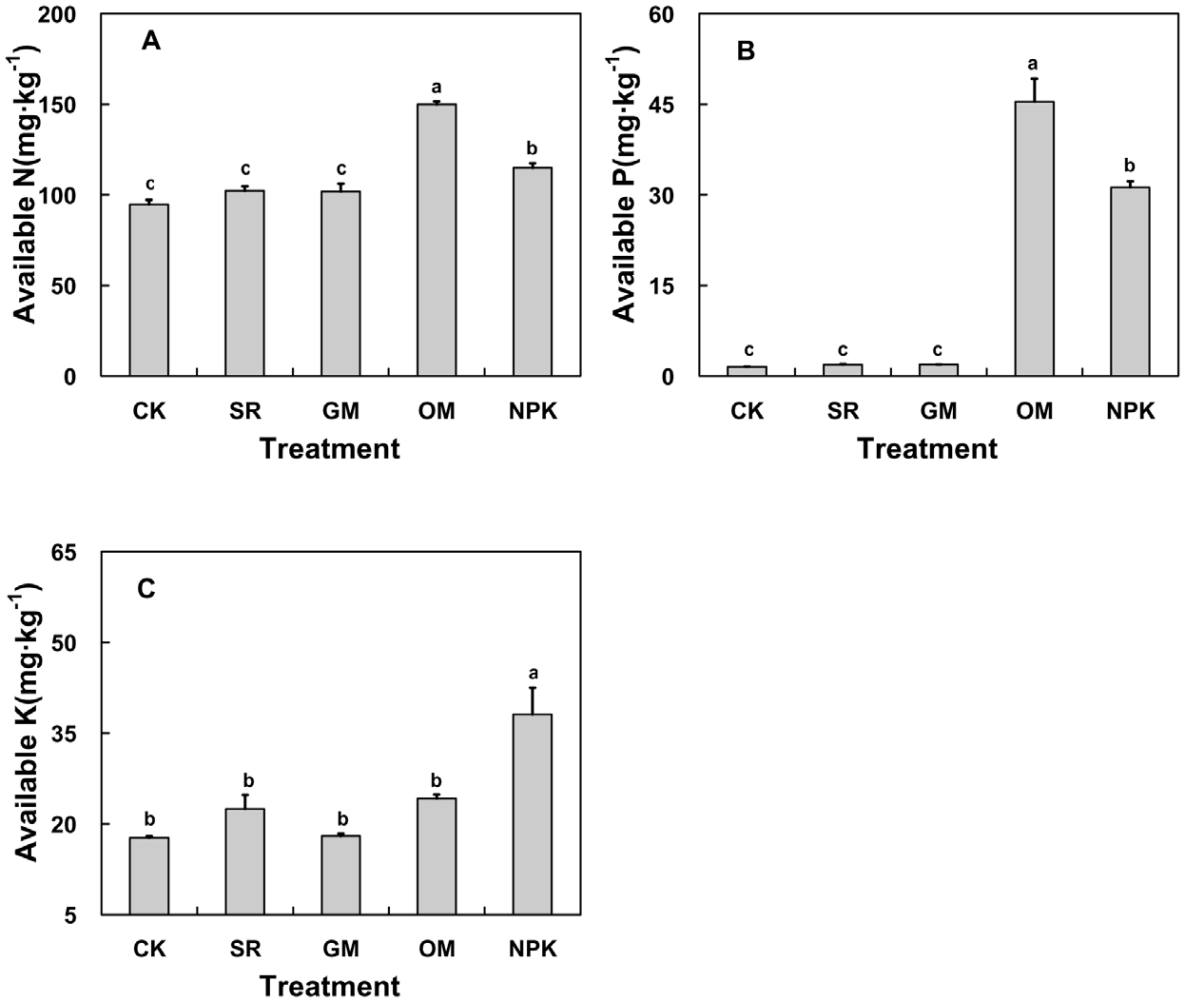
Average soil AN, AP and AK in the different fertilizer treatments. CK: no fertilizer; SR: straw returning rice field; NPK: chemical fertilizer; OM: organic manure and GM: green manure.

Soil physical and chemical properties were measured using the methods described by Bao [40]. Soil pH was measured with glass electrode in a 1∶2.5 soil/water suspension. SOC was measured by a K_2_CrO_7_-H_2_SO_4_ oxidation procedure and TN by the Kjeldahl method. Soil C/N values were calculated as the ratio SOC to TN. AN was determined by using a micro-diffusion technique after alkaline hydrolysis. AP was determined by the Olsen method. AK was measured by flame photometry after NH_4_OAc neutral extraction.

**Figure 8:**
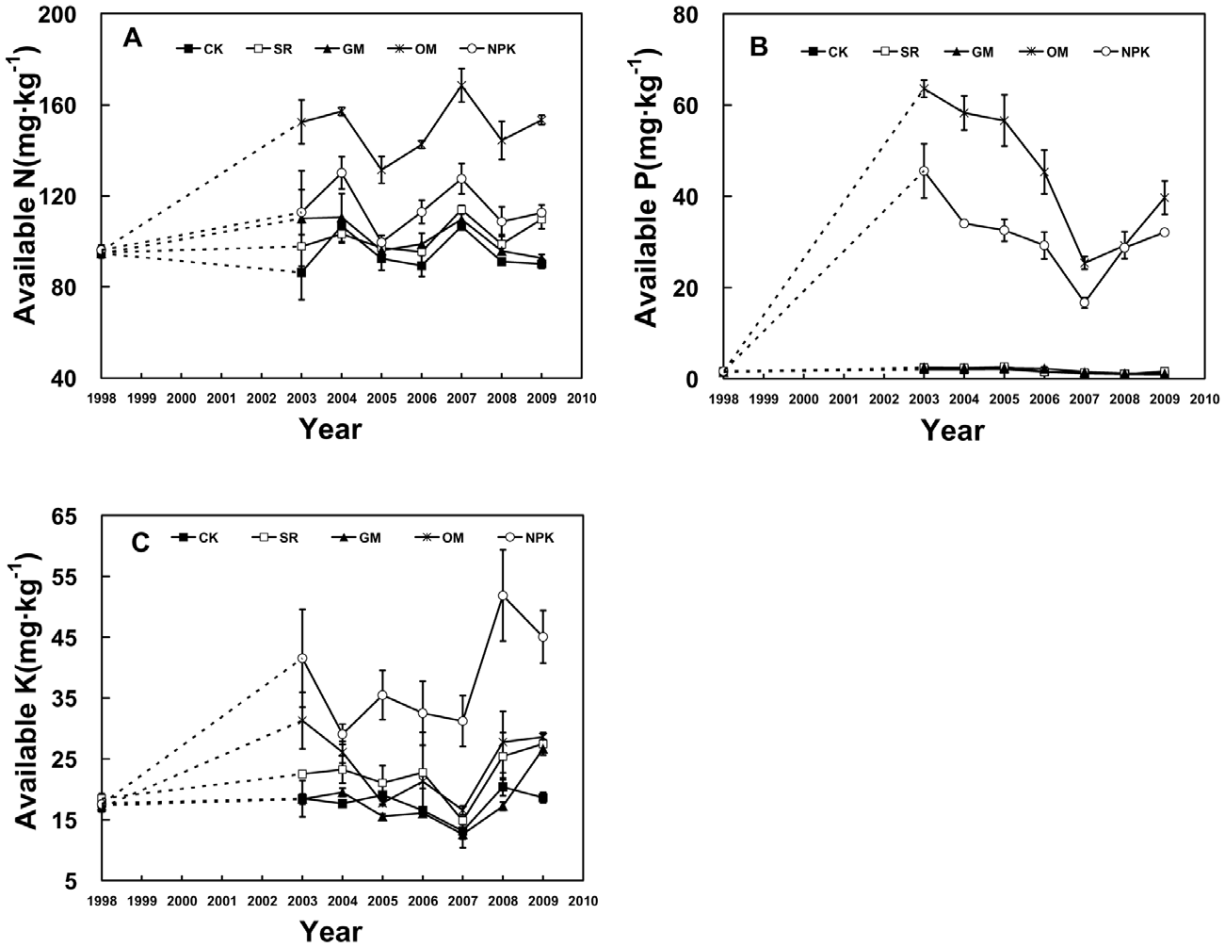
Dynamics of soil AN, AP and AK in the different fertilizer treatments during 1998–2009. soil samples for 1999–2002 were not analyzed (dashed lines).

### Data Analyses

All results were reported as means ± standard error (SE) for three replicates. One-way of variance (ANOVA) and Duncan’s multiple comparisons were performed to determine the differences among the fertilizer treatments in terms of the long-term soil nutrient contents means (during 2003–2009). Then the annual values under different treatments were used to investigate the dynamics of nutrient contents during the whole period. All statistical analyses were performed using the SPSS software package (version 15.0) (Statistical Graphics Crop, Princeton, USA). A difference at *P*<0.05 level was considered as statistically significant.

## Results

### Effects of Different Fertilizer Treatments on Soil pH

The average soil pH was shown in Fig.1. Statistical analysis revealed that fertilization treatments led to a significant increase in soil pH compared with the CK treatment (*P*<0.05). The soil pH was the lowest in CK with an average of 5.33 units and highest in NPK treatment with 5.89 units. In OM. treatment, the soil pH was relatively higher than CK (reaching 5.63 units).

During 1998 to 2009, the soil pH in NPK treatment appeared relatively stable despite some slight drop with the time (Fig.2). However, the values in other treatments showed a clear decline trend with time despite some variations. In the beginning years after fertilization, there was no evident difference in pH among all treatments, but eventually the soil pH in CK reduced dramatically and declined sharply from 5.71 to 5.03 (0.68 units lower). In SR, GM and OM treatments, the soil pH values declined by 0.57, 0.57 and 0.27 units respectively during the experimental period.

### Effects of Different Fertilizer Treatments on Soil Organic C and Total N

The SOC and TN contents showed statistically significant differences among the five treatments (Fig.3). We observed that the application of fertilizers (especially OM and NPK fertilizers) had remarkably improved SOC and TN values compared with the CK. Specifically, the OM treatment resulted in the highest SOC and TN concentrations (16.93 and 1.54 g·kg^−1^, respectively), which was 72.5%. and 51.2%^1^ higher than that of CK. The SOC and TN in NPK treatment were significantly higher than CK, reaching 11.97 and 1.20 g·kg^−1^, respectively. While in SR and GM treatment, the SOC was remarkably higher than CK, reaching 10.94 and 10.64 g·kg^−1^ respectively, but significant differences in TN contents between GM and CK were not observed.

The SOC in different treatments had a similar trend over time (Fig.4A). From 1998 to 2004, the SOC showed a clear increase with time due to fertilization, rising from initial 9.65–9.78 g·kg^−1^ to 11.51–20.00 g·kg^−1^ in 2004, respectively. Then SOC content dropped sharply but quickly reached at stable level. It was also obtained that the SOC content in OM was obviously higher than the other treatments during the experiment period, whereas that in CK remained relative stable (about 10 g·kg^−1^).

The dynamics of TN content in the five treatments followed similar patterns with SOC during 1998–2009 (Fig.4B). In the first few years, TN content tended to increase rapidly in the OM treatment (from 1.01 to 1.76 g·kg^−1^), followed by NPK treatment (from 1.02 to 1.33 g·kg^−1^). Thereafter, both of them declined and then maintained a certain level. Meanwhile, the soil TN contents in SR, GM and CK treatments were relatively steady, at approximately 1.05 g·kg^−1^.

### Effects of Different Fertilizer Treatments on Soil C/N Ratio

There were marked differences in soil C/N ratio among different treatments due to fertilizer application (Figure5). The average C/N ratio in the OM treatment (10.98) was obviously higher than the other treatments (*P*<0.05, Fig.5). Similarly, in SR treatment, the C/N ratio was significantly higher than CK. Nevertheless, there were no significant differences of C/N ratios in the CK, SR, GM and NPK treatments, ranging from 9.66 to 10.00.

Dynamics of soil C/N ratios during 1998–2009 are shown in Fig.6. From 1998 to 2003, the C/N ratios increased sharply in OM and GM treatments (25.0%and 17.9% higher than the initial amount), and then the values declined slowly and constantly till 2007 (reaching 10.14 and 8.98 respectively). However, the other treatments including CK, SR and NPK fluctuated at a stable level (approximately 10.0) from 1998 to 2007. In the last two years of the experiment period, all the five treatments displayed similar trends without a significant difference varying slightly between 8.98 and 11.29.

### Effects of Different Fertilizer Treatments on Soil Available Nutrients

A comparison of available nutrients among the treatments indicated that the fertilizer had a notable influence on soil AN, AP and AK (*P*<0.05, Fig.7A–C). During the experiment period, the average AN and AP contents in OM were highest (about 1.6 and 29.6 times of the CK, respectively) and second was NPK treatment (about 1.2 and 20.3 times of the CK). However, there were no obvious differences of AN and AP between SR, GM and CK treatments. Unlike AN and AP, the highest value of AK content was found in NPK treatment, which was 38.10 mg·kg^−1^ (about 2.2 times of the CK), and there were no obvious differences among the other four treatments (Fig.7C).

AN in the OM treatment obviously increased with time due to fertilization at the beginning, and then the value tended to rise with a slight fluctuation before remaining at the highest level in comparison to the other treatments. We also found a similar trend in the NPK treatment but lower than OM and did not find the significant differences between SR, GM and CK (Fig.8A).

During the entire experiment period, AP concentrations in CK, SR and GM treatments remained at an extremely low level (approximately 1.80 mg·kg^−1^) and were almost the same as the initial values. On the contrary, AP in both OM and NPK treatments displayed similar changes over time. Specifically, the value rose sharply in the first few years of fertilization (63.59 and 45.54 mg·kg^−1^ respectively in 2003) then progressively reduced till 2007 (25.37 and 16.68 mg·kg^−1^ respectively) and later increased slightly (Fig.8B).

The NPK treatment significantly increased AK content, especially from 2007 to 2009. However, the significant changes of AK in other treatments with time were not observed, maintaining at a stable and low level. (Fig.8C).

## Discussion

Many experiments have been conducted on the relationship between fertilization and soil pH [35], [41]. Some studies demonstrated that the soil pH was decreased to a certain extent with different fertilizer treatments [6]. In our study, the soil pH tended to drop in different treatments with time (Fig.2), and the decline rate in the NPK and OM treatments were relatively lower than CK, but producing higher pH values in the NPK and OM treatments than in the CK (Fig.1), which suggested that chemical fertilizer and organic manure could alleviate soil acidification to some extent. It has been reported that the application of alkaline fertilizer (e.g. calcium magnesium phosphate fertilizer) would return some alkaline substance to soils and thus increase the soil pH [42]. In addition, the application of organic manure could improve soil acidity by increasing the soil organic matter, promoting the soil maturation, improving the soil structure, and enhancing the soil base saturation percentage, which is in line with Zhang [35] and Li [43]. Moreover, studies showed that the soil pH in CK was lower than the initial value, which indicated that the acid deposition could have a great influence on the soil acidification in this trial region [44]. Since a too high or too low pH is harmful to the crop growth, it might be a practicable measure to establish the proper range of soil pH through fertilizer use.

Soil organic matter is a key contributor to soil due to its capacity to affect plant growth indirectly and directly [45]. As SOC and TN constitute heterogeneous mixtures of organic substances, they are widely used as the main parameters for evaluating soil fertility [46]. Meanwhile, human activities such as fertilizer practices and cropping systems play a key role in the regulation of C and N contents in agricultural soils [47]. In our experiments, SOC and TN contents increased considerably in the fertilization treatments compared with CK, especially in OM and NPK treatments (Fig.3), suggesting that organic and chemical fertilizer are beneficial to the accumulation of soil organic matter and thus improves soil fertility. This may be because both the application of organic manure and chemical fertilizer can improve soil aggregation, soil water retention, and reduce bulk density of the soil in the plough layer, promoting crop growth and the return of more root residues to the soil [48]. Under the SR application, SOC content was increased gradually with time, suggesting that the continuous SR supply had a positive effect in sustaining SOC, in accordance with the finding of Nie [49]. In farmland ecosystems, pig manure is easy to accumulate in soil and has lower ammonia losses than other fertilizers [33], so the TN concentration was significantly increased by continual annual OM applications compared to the other treatments (Fig.4B).

Previous studies have pointed out that the soil C/N ratio plays a key role in mineralization and accumulation of SOC and TN contents and that a high C/N ratio generally slows the mineralization process [47], [50].As found by Zhang [51], the C/N ratio was obviously higher in OM applications than the other treatments attributed to the SOC was more markedly enriched relative to TN. Moreover, we observed that there was a slight rise in soil C/N ratios with time in SR and NPK treatments (Fig.6), suggesting both SOC and TN accumulated gradually with time and the increase in SOC buildup was more rapid than TN over the past years in SR and NPK treatments [52].

It is known that fertilization is crucial for maintaining soil available nutrient levels, because fertilization ensures the largely constant presence of active microorganisms and the regular dynamic of biomass carbon [53]. Our research also showed that soil available nutrient contents were significantly affected by different fertilization treatments. Long-term OM application led to significantly higher values of soil AN and AP, compared to the other fertilization treatments (Fig.8A and 8B). It has also been reported by Huang [25] that significant AN and AP increases were observed in the manure-applied treatments. In addition, the AN and AP contents were maintained at a very higher level in the NPK treatments (Fig.8C) as a result of the long-term high inputs of N and P fertilizers [54], whereas AN and AP did not show statistically significant differences between CK, SR and GM treatments. Unlike AN and AP, AK was the highest in NPK treatments in our results, followed by the OM treatments, demonstrating that the K supplement from chemical and manure fertilizers are important and this may be the most advantageous way to solve the problem in China, where K resources are quite limited. Previous studies have shown that application of rice straw significantly increased available K while increasing organic matter contents [54], [55]. Similarly, the AK in SR treatment was higher than CK in our study. It was noted that the SOC, TN, C/N ratios and available nutrients had no significant difference under GM treatment in our study. Previous study had described that the decomposition process of green manure (as a fresh organic matter) was very slow and complicated, affected by soil temperature, moisture, plough back time and so on [56]. Hence, the real mechanism of the nutrient release of green manure deserves further study in order to make better use of green manure and increase its fertilizer efficiency in our study region.

Considering the dynamic changes of soil available nutrient content, we found that AN significantly increased with time from 1998 to 2009 in OM and NPK treatments, suggesting that the long-term soil organic matter played a major role in releasing soil AN. The evident increase of AK in NPK treatment over time in our study, suggests that continuously applying K fertilizer would dramatically improve the soil AK supply, In addition, AP values in both OM and NPK treatments were increased greatly compared to the other three treatments during the whole experiment period. This is consistent with many previous studies showing the accumulation of P is the most obvious and long-term manure or inorganic fertilizer application can significantly alter the amounts and proportion of labile and stable soil P pools [57]–[59]. That the OM treatment resulted in even higher AP value than NPK, also support the view that organic fertilizer is much more conducive to soil P availability rather than commercial P fertilizers in cropping systems that receive predominantly organic P amendments [59].

In conclusion, significant differences in soil fertility of paddy soils in the red soil region of southern China among different fertilization treatments were found in our study. Application of OM and NPK resulted in a substantial increase of SOC, TN, C/N ratios, AN and AP contents relative to the other fertilization treatments. Thus, it is likely that the OM and NPK application improves soil fertility. Meanwhile, the application of NPK would increase soil AK, leading to the highest AK contents. Continuous application of SR also had a positive effect in sustaining SOC, TN and C/N ratios. However, the effect of GM application on soil fertility was not remarkable compared to CK. Hence, organic manure should be recommended to improve soil fertility in this region and K fertilizer should be simultaneously applied considering the soil K contents. Moreover, in terms of the long-term fertilizer efficiency, annual straw returning application year by year could be adopted in this trail region.

## Supporting Information

Table S1 Data statistic and analysis for samples in this study. The detailed data of soil nutrient contents in different fertilization treatments for each samples in this study is shown.
(XLS)
